# High-Performance FAU Zeolite Membranes Derived from Nano-Seeds for Gas Separation

**DOI:** 10.3390/membranes13110858

**Published:** 2023-10-26

**Authors:** Qing Wang, Huiyuan Chen, Feiyang He, Qiao Liu, Nong Xu, Long Fan, Chuyan Wang, Lingyun Zhang, Rongfei Zhou

**Affiliations:** 1 School of Energy, Materials and Chemical Engineering, Hefei University, Hefei 230601, China; chenhuiyuan23@163.com (H.C.); hfy11121617@163.com (F.H.); liuqiao@hfuu.edu.cn (Q.L.); xunong@hfuu.edu.cn (N.X.); fanlong@hfuu.edu.cn (L.F.); lingyunf@hfuu.edu.cn (L.Z.); 2 State Key Laboratory of Materials-Oriented Chemical Engineering, College of Chemical Engineering, Nanjing Tech University, Nanjing 210009, China; 3 School of Biological Food and Environment, Hefei University, Hefei 230601, China; wangchuyan19@163.com

**Keywords:** FAU zeolite membrane, molecular sieve membrane, secondary growth, gas permeation, propylene propane separation, H_2_/C_3_H_8_ separation

## Abstract

In this study, high-performance FAU (NaY type) zeolite membranes were successfully synthesized using small-sized seeds of 50 nm, and their gas separation performance was systematically evaluated. Employing nano-sized NaY seeds and an ultra-dilute reaction solution with a molar composition of 80 Na_2_O: 1Al_2_O_3_: 19 SiO_2_: 5000H_2_O, the effects of synthesis temperature, crystallization time, and porous support (α-Al_2_O_3_ or mullite) on the formation of FAU membranes were investigated. The results illustrated that further extending the crystallization time or increasing the synthesis temperature led to the formation of a NaP impurity phase on the FAU membrane layer. The most promising FAU membrane with a thickness of 2.7 µm was synthesized on an α-Al_2_O_3_ support at 368 K for 8 h and had good reproducibility. The H_2_ permeance of the membrane was as high as 5.34 × 10^−7^ mol/(m^2^ s Pa), and the H_2_/C_3_H_8_ and H_2_/*i*-C_4_H_10_ selectivities were 183 and 315, respectively. The C_3_H_6_/C_3_H_8_ selectivity of the membrane was as high as 46, with a remarkably high C_3_H_6_ permeance of 1.35 × 10^−7^ mol/(m^2^ s Pa). The excellent separation performance of the membrane is mainly attributed to the thin, defect-free membrane layer and the relatively wide pore size (0.74 nm).

## 1. Introduction

The high energy consumption of gas separation has become one of the important environmental issues due to the seriousness of global climate change because the increase in traditional energy consumption will lead to an increase in CO_2_ emissions. Sholl et al. pointed out that the separation of alkenes from alkanes is one of the “seven chemical separations to change the world” [1]. The separation and purification of propylene and ethylene alone account for 0.3% of global energy use. Among them, propylene has long been one of the important chemical raw materials due to the wide application of its downstream products in the chemical and petroleum industries. Currently, propylene/propane separation is mainly performed by cryogenic distillation. However, separating propylene from propane is a great challenge since the boiling points of propylene (225.7 K) and propane (231 K) are close and the distillation process requires a large tower with 150–300 stages and a high reflux ratio [2]. The significant energy requirements and capital costs of cryogenic distillation processes have prompted research into other methods for separating these gases. Membrane-based gas separation is an attractive and forward-looking technology because it can be performed under mild conditions without phase changes and can be completed in one step, which can significantly reduce energy consumption by up to 80%, with a related decrease in the carbon footprint [3,4].

Various membrane materials, such as polymer membranes [5,6], zeolite membranes [3,7,8], metal-organic framework (MOF) membranes [9,10], mixed matrix membranes [11,12], and silicon carbide membranes [13,14,15], have been explored for separation. Among them, zeolites have become promising candidates for high-performance membranes in separation processes, catalytic membranes, and sensors considering their well-defined pore sizes, molecular sieving performance, high thermal stability, and high mechanical strength [16]. To date, much effort has been devoted to developing fine zeolite membranes. The FAU-type zeolite (including NaX with Si/Al ratio ≤ 1.5 and NaY with Si/Al ratio > 1.5) membranes with a pore diameter of 0.74 nm are suitable for separating large molecules that cannot be effectively handled by MFI (0.55 nm), LTA (0.42 nm), and CHA (0.38 nm) zeolite membranes. FAU membranes have been used in liquid separation (pervaporation), gas separation, and ion removal [17]. Combining the large pore size and affinity of FAU zeolites endows the membrane with high flux and selectivity [18].

So far, two typical methods for synthesizing supported FAU membranes have been developed [19]. The first method is in situ growth; that is, the porous support is immersed in the synthesis solution and then the required membrane layer is grown directly on the surface of the support through hydrothermal synthesis. Although this method is very simple, forming a pure phase and dense FAU membrane is difficult due to the poor heterogeneous nucleation of FAU crystal nuclei on the support surface during the in situ growth process [20]. The second widely used strategy for FAU membrane synthesis is secondary growth. Secondary growth includes depositing seed crystals on the support surface via rub coating, dip coating, or vacuum suction, followed by secondary hydrothermal synthesis [21]. Secondary growth has many advantages compared to in situ crystallization methods. It facilitates the control of the membrane microstructure (e.g., thickness and orientation). In particular, the seed layers can weaken the impact of the support, reduce defects, and achieve high reproducibility [18].

Over the past decades, FAU membranes have received extensive attention from researchers [22]. Shrestha et al. synthesized a continuous FAU membrane on a porous polyethersulfone support via secondary growth; the membrane exhibited a selectivity of 2.4 ± 0.8 for C_3_H_6_/C_3_H_8_ with a C_3_H_6_ permeance of (4.05 ± 1.86) × 10^−7^ mol/(m^2^ s Pa). Gu et al. synthesized dense FAU membranes on porous α-alumina (α-Al_2_O_3_) support using FAU seeds with 1–1.5 µm sizes [23]. The membrane had a thickness of 4 µm and exhibited CO_2_ selectivity with a separation factor of 31.2 for the CO_2_/N_2_ dry gas mixture and a CO_2_ permeance of 2.1 × 10^−8^ mol/(m^2^ s Pa). Zhou et al. prepared an FAU membrane with a thickness of ~2.3 µm on polydopamine (PDA)-modified α-Al_2_O_3_ support through in situ growth [19]. The membrane showed H_2_/CH_4_ and H_2_/C_3_H_8_ separation factors of 9.9 and 127.7, respectively, and exhibited H_2_ permeance as high as 1.9 × 10^−7^ mol/(m^2^ s Pa). However, the support modification process was relatively complex and the expensive PDA further limited the large-area preparation of the membrane. Recently, Nazir et al. systematically investigated the effect of FAU seed size (0.75–5.5 µm) on the formation of FAU membranes on α-Al_2_O_3_ supports [22]. Their results showed that the growth of the membrane layer was enhanced and the membrane defects were successfully reduced by using smaller seed particles of 750 nm, which is attributed to the minimal gaps between the seed particles. However, the size of the seeds (750 nm) was still too large to obtain high-quality, defect-free FAU membranes. Furthermore, an impurity phase NaP is easily formed in the zeolite layer during FAU membrane synthesis, making it difficult to prepare a pure-phase FAU membrane.

As mentioned above, the synthesis of high-performance FAU zeolite membranes still faces challenges such as the formation of intercrystalline defects, impure phases, and poor reproducibility. Many parameters can influence the formation of FAU membranes, such as gel composition, aging conditions, hydrothermal temperature, and time, as well as easily overlooked support properties and seed size. Understanding the influence of these parameters is important for controlling the synthesis process and producing high-performance FAU membranes. This work used nano-sized FAU seeds to explore the effects of crucial parameters (i.e., hydrothermal temperature and time) on the formation of FAU membranes on α-Al_2_O_3_ and mullite supports, respectively. High-performance FAU membranes were prepared by carefully tuning synthesis parameters to obtain optimal formation conditions. The as-prepared membranes exhibited excellent separation performance for H_2_/C_3_H_8_ and C_3_H_6_/C_3_H_8_.

## 2. Experimental

### 2.1. Materials

**Figure 1:**
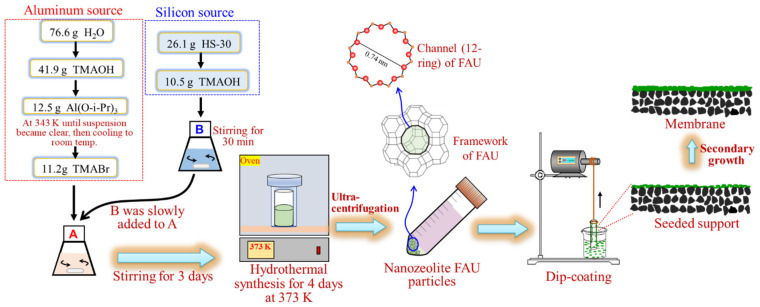
Schematic illustrations of the hydrothermal synthesis of nano-sized FAU seeds and membranes.

All procedures were performed under an ambient atmosphere unless otherwise stated. All reagents were obtained from commercial suppliers and used without further purification: Ludox HS-30 colloidal silica (SiO_2_/Na_2_O = 90, 30 wt% suspension in H_2_O, Aldrich), tetramethylammonium hydroxide (TMAOH, 25 wt% in H_2_O, Aldrich, Shanghai, China), aluminum isopropoxide (Al(O-i-Pr)_3_, 98 wt%, Aldrich, Shanghai, China), tetramethylammonium bromide (TMABr, 99 wt%, Aladdin, Shanghai, China), sodium aluminate (AlNaO_2_, Al/NaOH = 0.79, Wako, Osaka, Japan), sodium hydroxide (NaOH, 97 wt%, Aladdin, Shanghai, China), sodium silicate solution (reagent grade, SiO_2_ = 26.5 wt%, Na_2_O = 10.6 wt%, Aldrich, Shanghai, China), porous α-Al_2_O_3_ (average pore diameter: 200 nm, outer diameter: 12 mm, length: 6 cm), and mullite (average pore diameter: 1.3 μm, outer diameter: 12 mm, length: 6 cm) tube supports were provided by Nanjing Tech University. The preparation procedures for nano-sized NaY seeds and Nay-type zeolite FAU membranes are summarized in Figure 1.

### 2.2. Synthesis of Nano-NaY Seeds

Nano-sized NaY seeds were synthesized according to the literature [24], with slight modifications. The silicon and aluminum sources were prepared separately before mixing. Typically, the molar composition of the silicon source was 0.048Na_2_O: 4.35SiO_2_: 0.96TMAOH: 48.46H_2_O; that is, 26.2 g Ludox HS-30 colloidal silica and 10.46 g TMAOH were mixed and stirred at room temperature for 30 min. The molar composition of the alumina source was 1Al_2_O_3_: 3.84TMAOH: 2.4TMABr: 200.54H_2_O. 76.6 g H_2_O and 41.9 g TMAOH were mixed and 12.5 g Al(O-i-Pr)_3_ was dissolved in this mixture while stirring in a water bath at 343 K until the suspension became clear. After cooling to room temperature, 11.2 g TMABr was added to the mixture and stirred at room temperature for 15 min. Then, the silica source was slowly added to the aluminum source while stirring. The final molar composition of the nano-sized NaY synthesis solution was 0.048Na_2_O: 4.80TMAOH: 2.4TMABr: 4.35SiO_2_: 1.0Al_2_O_3_: 249H_2_O. The clear solution was aged by stirring at room temperature for 3 days, and then transferred into a Teflon-lined autoclave and heated in an oven at 373 K for 4 days. In the product mixture, nano-sized NaY particles were captured using an ultracentrifuge and washed with distilled water until pH 7. Finally, the seeds were dispersed in water to obtain a 1 wt% seed suspension.

### 2.3. Synthesis of FAU (NaY) Membrane

FAU (NaY) zeolite membranes were hydrothermally synthesized on the outer surface of tube supports. As reported previously [3], the NaY seeds were coated on the outer surface of the tubular support via a dip-coating technique. Before seeding, both ends of the tubular support were sealed with silicone plugs to prevent the inner surface from coating with seeds. The dried tubular supports were immersed in the seed suspension for 60 s and subsequently dried at 373 K for 15 min.

The molar composition used for membrane growth was 80Na_2_O: 1Al_2_O_3_: 19SiO_2_: 5000H_2_O; the specific procedure followed is described below. AlNaO_2_ was added to a mixed solution of deionized water and NaOH while stirring at room temperature for 0.5 h. Sodium silicate solution was added to the above solution while stirring for 12 h, then transferred into a Teflon-lined autoclave. The seeded tubular supports were placed vertically in an autoclave and fully immersed in the gel. Hydrothermal synthesis was done in an oven at 358–378 K for 3–10 h. After crystallization, the synthesized membranes were removed from the solution, washed with water, and dried at 333 K.

### 2.4. Characterization and Gas Permeation

The morphology and Si/Al ratio of seeds and membranes were observed using a Hitachi S-4800 scanning electron microscope (SEM) coupled with an energy-dispersive X-ray (EDX) analyzer. The crystalline phases of seeds and membranes were identified by X-ray diffraction (XRD, Ultima IV) using Cu Kα radiation in the 2*θ* range from 5 to 45°. Before gas permeation measurement, the membrane was placed in a vacuum oven at 373 K overnight to remove water from the zeolite pores. Single-gas permeation performance of FAU membranes was evaluated at room temperature using an experimental setup schematically shown elsewhere [25,26]. Single gases (H_2_, 0.289 nm; CO_2_, 0.33 nm; N_2_, 0.364 nm; CH_4_, 0.38 nm; C_3_H_6_, 0.47 nm; C_3_H_8_, 0.51 nm, and i-C_4_H_10_, 0.53 nm) with different kinetic diameters were fed to the outside of the cylindrical membrane. The feed gas was pressurized at ~0.4 MPa (except for i-C_4_H_10_, which was pressurized at 0.1 MPa), while the permeate stream was maintained at atmospheric pressure. The permeate flow rate was measured using a soap-film meter. The selectivity is defined as the permeance ratio of single gases [27,28].

## 3. Results and Discussion

### 3.1. Characterization of FAU Seed and Seed Layer

**Figure 2:**
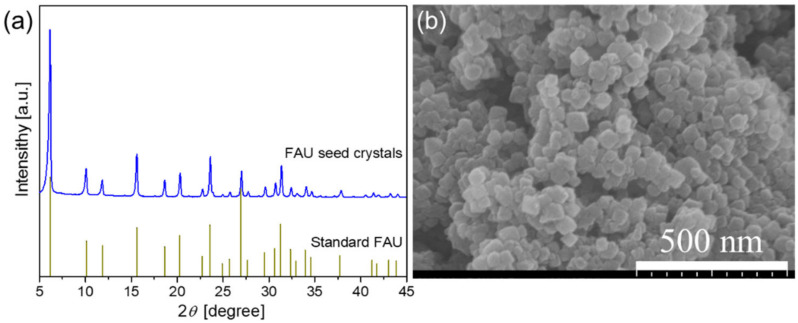
(**a**) XRD pattern and (**b**) SEM image of the as-synthesized FAU seeds.

Figure 2 shows the XRD pattern and the SEM image of the as-synthesized FAU seeds. The XRD pattern (Figure 2a) of the as-synthesized zeolite sample shows a series of characteristic peaks at 2θ = 6.2°, 10.1°, 15.58°, 18.62°, 23.54°, and 31.27°, which matched well with the standard FAU, indicating that the seed crystals were pure FAU. The SEM image (Figure 2b) shows that the zeolite particle morphology is roughly spherical, with an average particle size of approximately 50 nm. The Si/Al ratio of the seed crystals was 1.9 based on the EDX analysis, which is within the Si/Al range of NaY zeolite. Therefore, nano-sized NaY seeds with pure phase were successfully synthesized.

**Figure 3:**
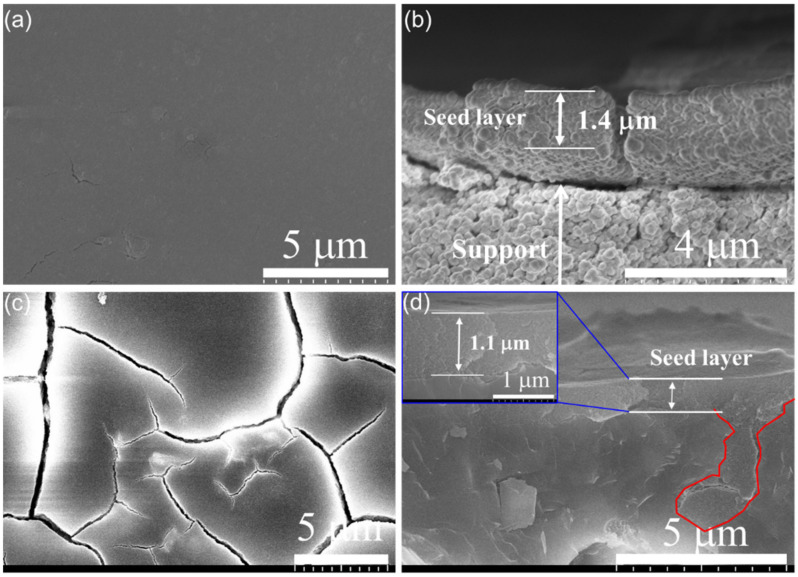
Surface (**a**,**c**) and cross-sectional (**b**,**d**) SEM images of seeded α-Al_2_O_3_ (**a**,**b**) and mullite supports (**c**,**d**). The seeds penetrating the mullite support and filling the internal pores are highlighted in red lines.

Some seeding methods have been used to prepare zeolite membranes, among which the dip-coating method is simpler. Figure 3 shows the surface and cross-sectional SEM images of the seeded α-Al_2_O_3_ and mullite supports using dip-coating. It was observed that the surface of the porous α-Al_2_O_3_ support was completely covered with a uniform, smooth, densely-packed FAU seed layer with a thickness of approximately 1.4 µm. The surface of the porous mullite support was also completely covered with a uniform FAU seed layer with a thickness of ~1.1 µm. However, the seed layer on mullite support exhibited obvious cracks, which could be attributed to the high roughness of the microporous (1.3 µm) mullite surface. In addition, it can be observed from the cross-sectional SEM images of the seeded supports that the seeds had not penetrated the internal pores of the α-Al_2_O_3_ support; on the contrary, the seeds penetrated the mullite support and filled the internal pores (highlighted in red line in Figure 3d), and there was no obvious boundary between the seed layer and the support. Different supports will impact the microstructure and properties of FAU membranes, as will be discussed later when describing the synthesis of the membranes.

### 3.2. Membrane Synthesis under Different Conditions

The preparation of zeolite membranes is affected by many factors, among which reasonable control of temperature and crystallization time is crucial for the preparation of pure FAU zeolite membranes and the avoidance of the formation of impurity phases. In this study, FAU membranes were prepared by hydrothermal synthesis at 358–378 K for 3–10 h by immersing the seeded supports in an ultra-dilute solution with a molar composition of 80Na_2_O: 1Al_2_O_3_: 19SiO_2_: 5000H_2_O. Compared with previous reports [19,23,29], the dilute clear reaction solution had a high water content, which plausibly reduced the nucleation effect in the bulk of the reaction solution and instead promoted crystal growth on the seed layer [21,30].

#### 3.2.1. Effect of Synthesis Time on Membrane Formation on α-Al_2_O_3_ Support

**Figure 4:**
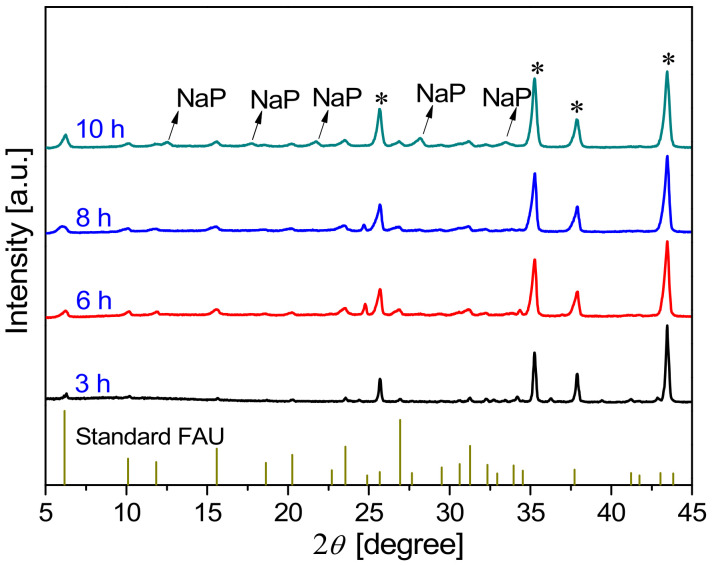
XRD patterns of FAU membranes prepared on α-Al_2_O_3_ supports at 368 K with different crystallization times (*: α-Al_2_O_3_ support).

Figure 4 shows the XRD patterns of FAU membranes synthesized at 368 K for 3–10 h. It can be observed that the membrane synthesized for 3 h showed weak characteristic peaks of FAU zeolite at 2*θ* values of 6.2°, 10.1°, 15.58°, 23.54°, and 31.27°, indicating that the crystallinity of the zeolite layer was low. The peak intensity of as-synthesized FAU membranes increased with synthesis time, indicating the growth of the zeolite layer. However, when the synthesis time was prolonged to 10 h, some weak peaks were observed at 2*θ* = 12.5°, 17.7°, 21.7°, 28.2°, and 33.4°, indicating that NaP zeolite existed in the membrane as an impurity phase [31]. The formation of NaP impurity when preparing the FAU zeolite membrane was similar to that of T-type zeolite when preparing LTA zeolite over a long synthesis time [32].

**Figure 5:**
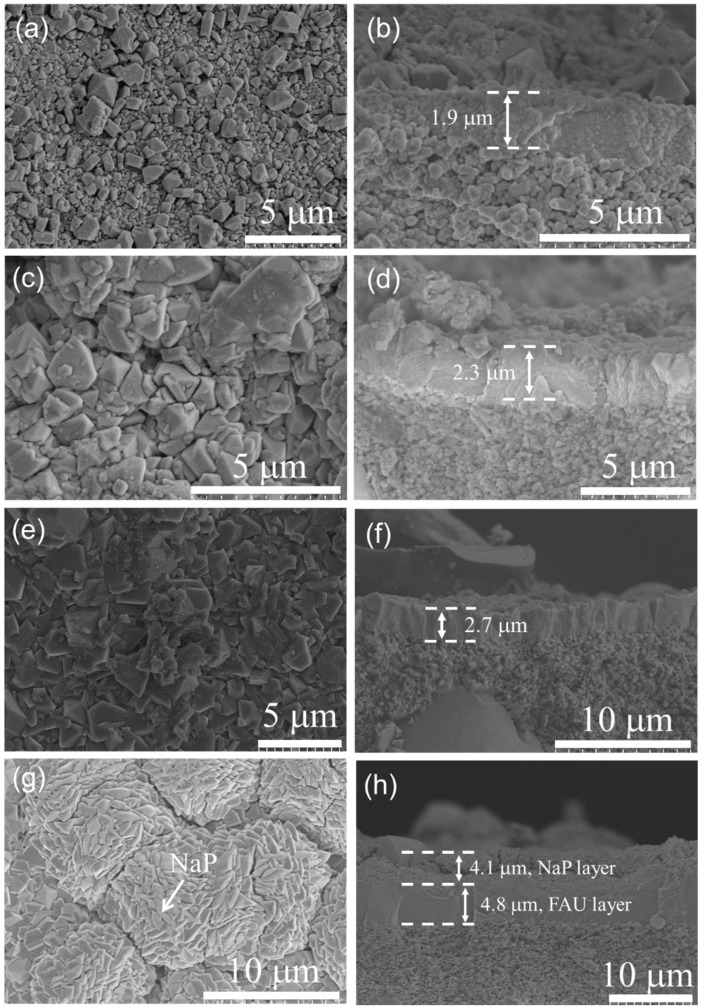
Surface and cross-sectional SEM images of FAU membranes synthesized at 368 K on α-Al_2_O_3_ supports with different crystallization times. (**a**,**b**) 3 h, (**c**,**d**) 6 h, (**e**,**f**) 8 h, (**g**,**h**) 10 h.

Figure 5 shows the SEM images of FAU membranes synthesized at 368 K for different synthesis times. It can be observed that the membrane synthesized for a crystallization period of 3 h had a loose membrane layer (Figure 5a) and the membrane thickness increased from a thick seed layer of 1.4 µm to ~1.9 µm (Figure 5b). Notably, the original seed crystals with a size of 50 nm in the seed layer grew larger (0.5–1 µm). Still, the crystal morphology was incomplete, indicating that it is not completely crystallized, which is consistent with the XRD results in Figure 4. When the synthesis time was increased to 6 h (Figure 5c,d), the facets (or outlines) of the crystals became slightly clear, but they were loosely connected to each other and the thickness of the membrane was approximately 2.3 μm. When the synthesis time was extended to 8 h (Figure 5e,f), a continuous, uniform, and dense zeolite layer was formed on the support and the thickness of the zeolite membrane was about 2.7 µm. When the crystallization time was prolonged to 10 h (Figure 5g,h), a large number of walnut-like crystals appeared on the surface of the zeolite layer. The walnut-like crystals were NaP zeolite, according to the XRD pattern (Figure 4). Therefore, the membrane was roughly divided into two layers: the bottom layer was an FAU zeolite layer with a thickness of ~4.8 μm and the top layer was a NaP layer with a thickness of ~4.1 μm. The presence of NaP crystals in the membrane layer could reduce the membrane permeability due to the smaller pore size of P zeolite (~0.29 nm) and reduce the thermal stability of the membrane due to the cubic-to-tetragonal phase transformation of NaP, even at low temperatures [23,33]. The Si/Al ratio of the membrane synthesized at 368 K for 8 h was ~2.1 as measured by EDX, indicating that the as-synthesized FAU membrane belongs to the NaY type. To avoid impurity phases, the subsequent investigations were conducted with a synthesis time of 8 h.

#### 3.2.2. Effect of Synthesis Temperature on Membrane Formation on α-Al_2_O_3_ Support

Crystallization temperature is an important parameter for forming a specific type of zeolite phase. The hydrothermal synthesis temperature affects the nucleation and crystallization of zeolites, with higher synthesis temperatures leading to higher energy, which is beneficial to the crystallization process [21].

**Figure 6:**
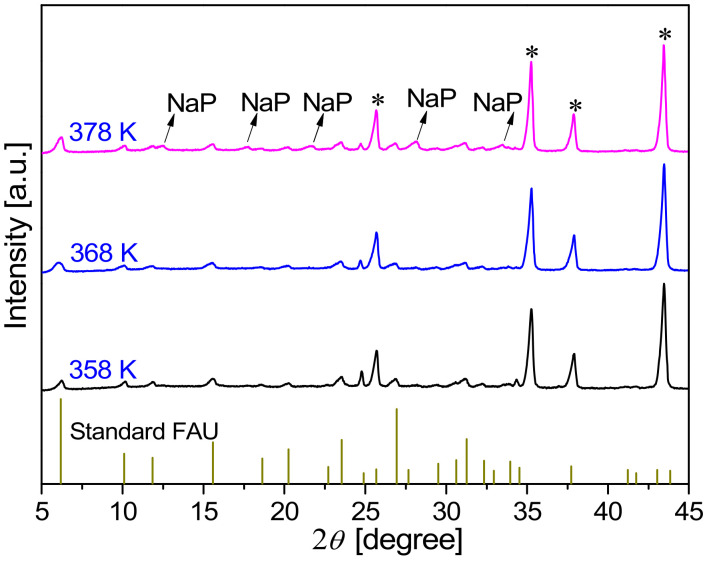
XRD patterns of FAU zeolite membranes prepared on α-Al_2_O_3_ supports for 8 h at different crystallization temperatures (*: α-Al_2_O_3_ support).

**Figure 7:**
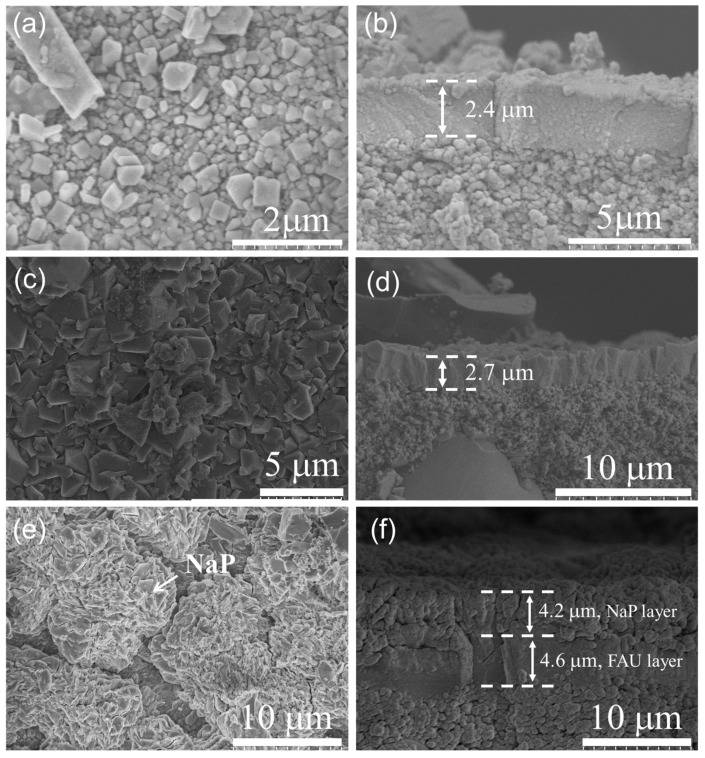
Surface and cross-sectional SEM images of FAU zeolite membranes synthesized on α-Al_2_O_3_ support for 8 h at different crystallization temperatures. (**a**,**b**) 358 K, (**c**,**d**) 368 K, (**e**,**f**) 378 K.

Figure 6 and Figure 7 show the XRD patterns and SEM images, respectively, of the membranes synthesized for 8 h at different temperatures. As shown in Figure 6, the intensity of the characteristic peaks of the synthesized membranes increased with the synthesis temperature. The membrane synthesized at 358 K for 8 h showed weak FAU characteristic peaks, indicating the formation of FAU zeolite on the support. However, loosely packed small crystals with incomplete shapes formed the membrane-like layer with a thickness of 2.4 µm on the α-Al_2_O_3_ support (Figure 7). The intensity of the characteristic peaks of the membranes synthesized at 368 K was significantly enhanced and a pure, fully covered, dense FAU layer was obtained on the α-Al_2_O_3_ support (as mentioned before). When the synthesis temperature increased to 378 K, the characteristic peaks of NaP appeared in the XRD pattern of the synthesized membrane. The membrane layer included an FAU layer with a thickness of ~4.6 μm and a NaP layer of ~4.2 μm. These results suggest that the crystallization rate increases with increasing synthesis temperature; however, the phase transition from the FAU-type zeolite structure to the NaP impurity zeolite structure becomes more significant [34].

#### 3.2.3. Effect of Synthesis Time on Membrane Formation on Mullite Support

Based on the details discussed thus far, employing FAU seeds with a size of 50 nm and an ultra-dilute reaction solution, the effects of synthesis temperature and time on the formation of FAU membranes on α-Al_2_O_3_ supports were investigated; the optimized conditions for membrane synthesis were determined to be 368 K for 8 h. In this section, we set the synthesis temperature at 368 K and tried to prepare satisfactory FAU membranes on cheap mullite supports by controlling the crystallization time, despite the different pore sizes of the mullite and α-Al_2_O_3_ supports.

**Figure 8:**
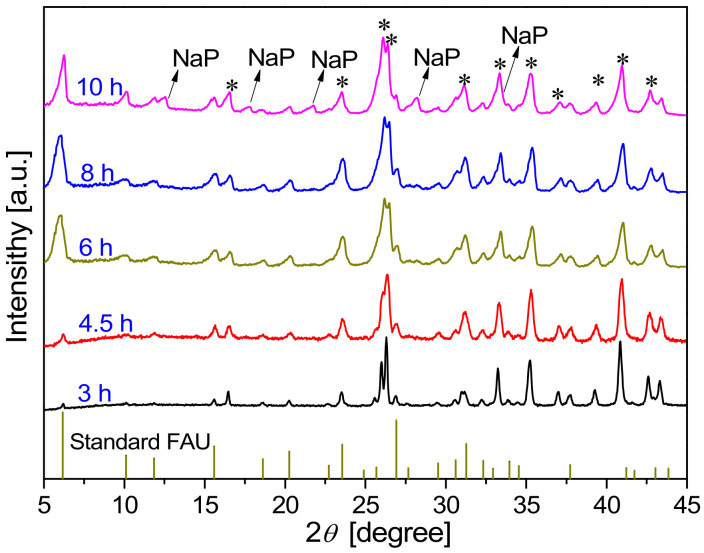
XRD patterns of FAU zeolite membranes synthesized on mullite supports at 368 K for different crystallization times (*: mullite support).

**Figure 9:**
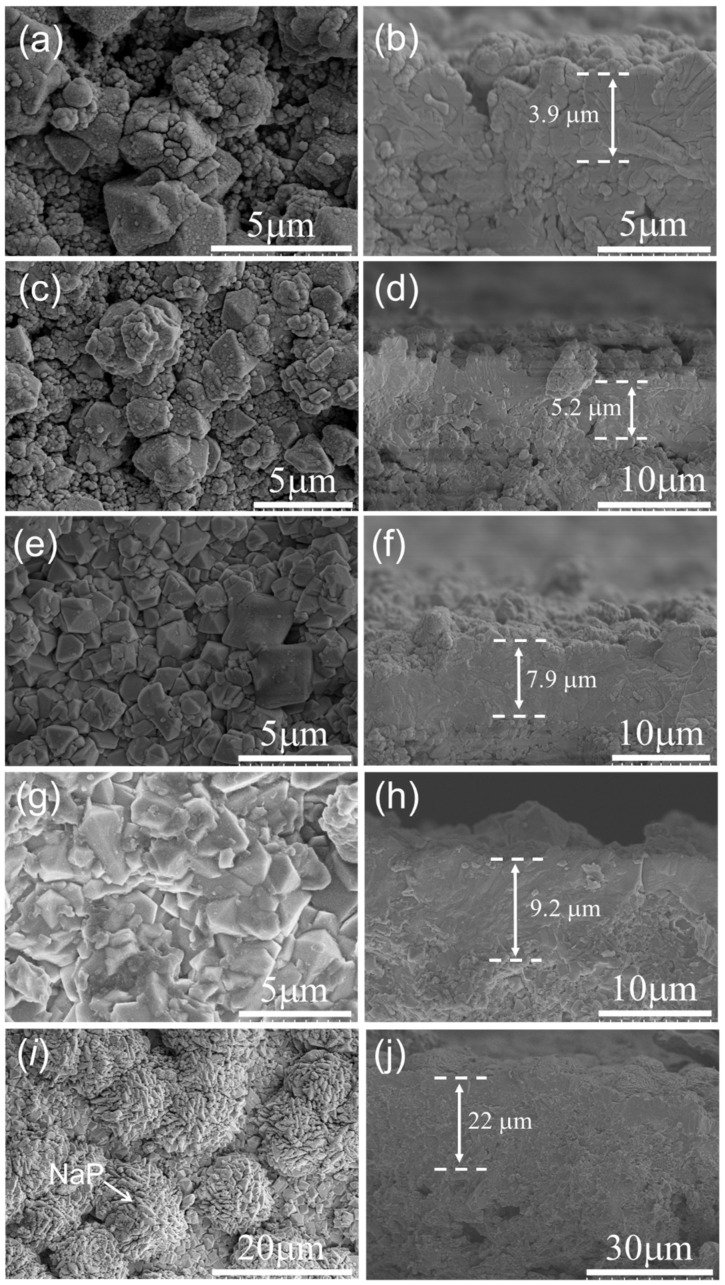
Surface and cross-sectional SEM images of FAU zeolite membranes with different crystallization times on mullite supports. (**a**,**b**) 3 h, (**c**,**d**) 4.5 h, (**e**,**f**) 6 h, (**g**,**h**) 8 h, (**i**,**j**) 10 h.

Figure 8 and Figure 9 show the XRD patterns and SEM images, respectively, of the membranes synthesized on mullite supports at 368 K for different crystallization times. As shown in Figure 8, the intensity of characteristic peaks of the synthesized membranes increased with the synthesis time. When the synthesis time increased from 3 to 4.5 h, the intensity of the characteristic peaks of FAU was slightly enhanced, the crystals on the membrane surface were incomplete, and the membrane thickness increased from 1.1 µm (the seed layer) to 5.2 µm (Figure 9a–d). This indicates that the growth of seeds from the seed layer and support pores occurred within 4.5 h of synthesis.

When the synthesis time increased from 4.5 to 6 h, the intensity of the characteristic peaks of FAU was significantly increased, while the increase from 6 to 8 h was only slight. Figure 9e,f shows that the membrane layer with a thickness of 7.9 μm was continuous and dense without obvious cracks and holes after 6 h of synthesis. The surface morphology of the membrane synthesized for 8 h was similar to that of the membrane synthesized for 6 h. The main difference was that after 8 h of synthesis, the membrane layer began to grow into the support, increasing the membrane thickness to ~9.2 μm (Figure 9g,h). However, a thick membrane layer increases mass transfer resistance and reduces permeability [35,36]. When the synthesis time was prolonged to 10 h, the characteristic peaks of the NaP impurity phase appeared. Figure 9i,j show that there were many walnut-like NaP crystals on the membrane surface and the growth of the membrane layer towards the interior of the support became more severe, resulting in the membrane thickness increasing to ~22 μm. The Si/Al ratio of the membrane synthesized on the mullite support at 368 K for 6 h was ~2.9 (NaY type) as measured by EDX; this membrane was selected as a promising FAU membrane for gas separation.

It is worth noting that membranes supported by mullite were thicker and had higher Si/Al ratios than membranes supported by α-Al_2_O_3_, which was plausibly attributed to the silicon element contained in mullite providing silicon-nutrient for the growth of FAU membranes.

### 3.3. Gas Separation Performance

Based on the characteristics described thus far, two types of FAU (NaY) membranes with continuous, uniform, and dense zeolite layers, that is, α-Al_2_O_3_-supported membranes (hereafter referred to as MA-*x*) synthesized at 368 K for 8 h and mullite-supported membranes (hereafter referred to as MM-*x*) synthesized at 368 K for 6 h, were selected as promising FAU membranes for gas separation.

#### 3.3.1. Gas Permeation

**Figure 10:**
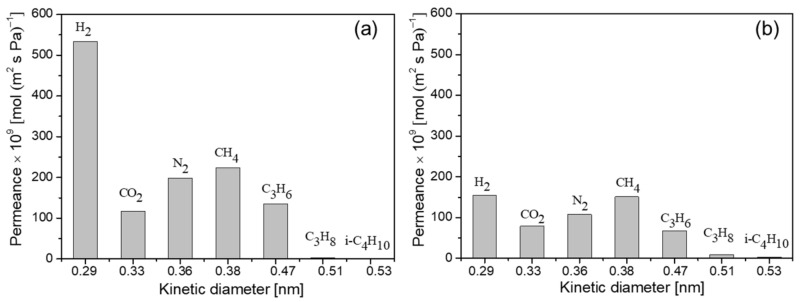
Single-gas permeance as a function of kinetic diameter at room temperature through (**a**) MA-1 (on the α-Al_2_O_3_ support) and (**b**) MM-1 (on the mullite support) membranes.

Figure 10 shows the single-gas permeance for H_2_, CO_2_, N_2_, CH_4_, C_3_H_6_, C_3_H_8_, and *i*-butane (*i*-C_4_H_10_) as a function of the kinetic diameter at room temperature through the MA-1 and MM-1 membranes. The permeances of these gases, except CO_2_ and N_2_, through both membranes decreased with the kinetic diameter of the molecules. The permeance order was H_2_ > CH_4_ > C_3_H_6_ > C_3_H_8_ > *i*-C_4_H_10_. This is because the permeation of small-sized molecules (e.g., H_2_, CO_2_, N_2_, and CH_4_) through the FAU (pore size: 0.74 nm) membrane is mainly controlled by Knudsen diffusion, which is inversely proportional to the square root of the molecular weight ratio of the gases. However, the behavior of large-sized molecules permeating the membrane results from the combined effects of Knudsen diffusion, affinity (e.g., C_3_H_6_), and molecular sieving (e.g., i-C_4_H_10_). The H_2_ permeance of the MA-1 membrane was as high as 5.34 × 10^−7^ mol/(m^2^ s Pa), which is 3.4 times that of the MM-1 membrane. In addition, the H_2_/C_3_H_8_ selectivity of the MA-1 membrane was 183, 10.1 times that of the MM-1 membrane and the H_2_/i-C_4_H_10_ selectivity was as high as 315, 6.8 times that of the MM-1 membrane. The results demonstrate that, compared to the MM-1 membrane, the MA-1 membrane layer was thinner and with no defects, reducing permeation resistance and maintaining high selectivity. It is worth noting that the C_3_H_6_/C_3_H_8_ selectivity of the MA-1 membrane was as high as 46 and was coupled with a remarkably high C_3_H_6_ permeance of 1.35 × 10^−7^ mol/(m^2^ s Pa). In this work, both types of synthesized membranes had excellent selectivity for propylene/propane; that is, the permeance of propylene was higher than that of propane. This is because the propylene molecule has a smaller dynamic diameter and there is a suitable electrostatic force between FAU (NaY) zeolite and the double bond of propylene, hence promoting propylene molecule permeation [37,38].

#### 3.3.2. Membrane Reproducibility

More than three FAU membranes were re-prepared for each type (i.e., MA and MM), and their gas separation performance was compared to confirm the reproducibility of the membranes, as listed in Table 1. Based on the examination of all the data, each type exhibited similar levels of permeability and selectivity. The relative standard deviations of permeance and selectivity were maintained within 14.8%, demonstrating that the membranes are reproducible [3].

#### 3.3.3. Comparing Gas Separation Performance with Data in the Literature

**Figure 11:**
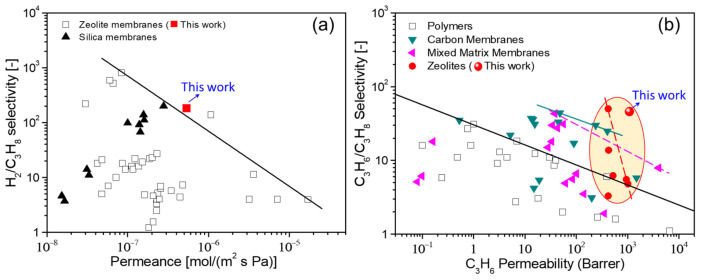
Comparing the separations of (**a**) H_2_/C_3_H_8_ and (**b**) C_3_H_6_/C_3_H_8_ for gas separation membranes (original data are shown in Tables S1 and S2).

Substantial efforts have been made to develop high-performance zeolite membranes for gas separation. The H_2_/C_3_H_8_ and C_3_H_6_/C_3_H_8_ separations are the most important and attractive processes in the petrochemical industry. For example, the products of direct dehydrogenation of propane to propylene (such as the Oleflex process) include abundant H_2_, C_3_H_6_, and C_3_H_8_ [39]. The membrane needs to have good H_2_/C_3_H_8_ and C_3_H_6_/C_3_H_8_ selectivity to achieve efficient separation and purification of the products. Figure 11 summarizes the separation performances of H_2_/C_3_H_8_ and C_3_H_6_/C_3_H_8_ using FAU (MA-1) membrane synthesized on the α-Al_2_O_3_ support together with the membranes reported in recent years, which include polymer, silica, carbon, mixed matrix, and zeolite membranes (details are shown in Tables S1 and S2). Obviously, the MA-1 membrane has demonstrated attractive separation performance for H_2_/C_3_H_8_ (H_2_ permeance = 5.34 × 10^−7^ mol (m^2^ s Pa)^−1^, selectivit = 183) and C_3_H_6_/C_3_H_8_ (C_3_H_6_ permeance = 1.35 × 10^−7^ mol (m^2^ s Pa)^−1^, selectivity = 46). It is worth mentioning that compared with various types of membranes, the C_3_H_6_/C_3_H_8_ separation performance of the MA-1 membrane far surpasses the upper boundaries of the “tradeoff” line. The impressive gas separation capability was attributed to the thin membrane layer, the defect-free, and the high quality of the as-prepared FAU membrane (the optical photograph of the membrane is shown in Figure S1).

## 4. Conclusions

In this study, high-performance FAU (NaY type) zeolite membranes were successfully synthesized using small-sized seeds of 50 nm, and their gas separation performance was evaluated. The results illustrated that further extending the crystallization time or increasing the synthesis temperature led to the formation of a NaP impurity phase on the FAU membrane layer. The most promising FAU membrane with a thickness of 2.7 µm was synthesized on an α-Al_2_O_3_ support at 368 K for 8 h and had good reproducibility. The H_2_ permeance of the membrane was as high as 5.34 × 10^−7^ mol/(m^2^ s Pa) and the H_2_/C_3_H_8_ and H_2_/i-C_4_H_10_ selectivities were 183 and 315, respectively. The most attractive performance was that the C_3_H_6_/C_3_H_8_ selectivity of the membrane was as high as 46, with a remarkably high C_3_H_6_ permeance of 1.35 × 10^−7^ mol/(m^2^ s Pa). The excellent separation performance of the membrane is mainly attributed to the thin, defect-free membrane layer and the relatively wide pore size.

## Figures and Tables

**Table 1:** Gas separation performance of FAU (MA and MM) membranes prepared under optimized conditions.

Membrane	Permeances [×10^−7^ mol (m^2^ s Pa)^−1^]		Selectivity		
	H_2_	C_3_H_6_	H_2_/C_3_H_8_	H_2_/i-C_4_H_10_	C_3_H_6_/C_3_H_8_
MA-1	5.34	1.35	183	315	46
MA-2	5.11	1.22	176	309	42
MA-3	4.82	1.01	188	324	39
MM-1	1.55	0.68	18	46	7.9
MM-2	1.81	0.79	15	39	6.5
MM-3	1.50	0.59	14	44	5.5
Average for MA	5.09 ± 0.21	1.19 ± 0.14	182 ± 5	316 ± 6	42.3 ± 2.87
Average for MM	1.62 ± 0.14	0.68 ± 0.08	16 ± 2	43 ± 3	6.6 ± 0.98

## Data Availability

Not applicable.

## Supplementary Materials

The following supporting information can be downloaded at: https://www.mdpi.com/article/10.3390/membranes13110858/s1, Table S1: Comparison of the H_2_/C_3_H_8_ separation properties of FAU membranes (MA and MM) with other membranes from recent literature; Table S2: Membrane separation performance data of C_3_H_6_/C_3_H_8_; Figure S1: The optical photograph of an FAU (MA) membrane. Refs. [40,41,42,43,44,45,46,47,48,49,50,51,52,53,54,55,56,57,58,59,60,61,62,63,64,65,66,67,68,69,70,71,72,73,74,75,76,77,78,79,80,81,82,83,84,85,86,87,88,89,90,91] are cited in Supplementary Materials.

## References

[1] Sholl D.S., Lively R.P. (2016). Seven chemical separations to change the world. Nature.

[2] Shrestha S., Dutta P.K. (2019). Modification of a continuous zeolite membrane grown within porous polyethersulfone with Ag (I) cations for enhanced propylene/propane gas separation. Microporous Mesoporous Mater..

[3] Wang Q., Wu A., Zhong S., Wang B., Zhou R. (2017). Highly (h0h)-oriented silicalite-1 membranes for butane isomer separation. J. Membr. Sci..

[4] Ren Y., Liang X., Dou H., Ye C., Guo Z., Wang J., Pan Y., Wu H., Guiver M.D., Jiang Z. (2020). Membrane-based olefin/paraffin separations. Adv. Sci..

[5] Sandru M., Sandru E.M., Ingram W.F., Deng J., Stenstad P.M., Deng L., Spontak R.J. (2022). An integrated materials approach to ultrapermeable and ultraselective CO_2_ polymer membranes. Science.

[6] Xia Y., Cao H., Xu F., Chen Y., Xia Y., Zhang D., Dai L., Qu K., Lian C., Huang K. (2022). Polymeric membranes with aligned zeolite nanosheets for sustainable energy storage. Nat. Sustain..

[7] Wang N., Dang G., Bai Z., Wang Q., Liu B., Zhou R., Xing W. (2023). In Situ Synthesis of Cation-Free Zirconia-Supported Zeolite CHA Membranes for Efficient CO_2_/CH_4_ Separation. ACS Appl. Mater. Interfaces.

[8] Huang W., He Z., Liu B., Wang Q., Zhong S., Zhou R., Xing W. (2023). Large surface-to-volume-ratio and ultrahigh selectivity SSZ-13 membranes on 61-channel monoliths for efficient separation of CO_2_/CH_4_ mixture. Sep. Purif. Technol..

[9] Wei R., Liu X., Zhou Z., Chen C., Yuan Y., Li Z., Li X., Dong X., Lu D., Han Y. (2022). Carbon nanotube supported oriented metal organic framework membrane for effective ethylene/ethane separation. Sci. Adv..

[10] Basel N., Liu Q., Fan L., Wang Q., Xu N., Wan Y., Dong Q., Huang Z., Guo T. (2022). Surface charge enhanced synthesis of TpEB-based covalent organic framework (COF) membrane for dye separation with three typical charge properties. Sep. Purif. Technol..

[11] Liu Q., Basel N., Li L., Xu N., Dong Q., Fan L., Wang Q., Ding A., Wang T. (2022). Interfacial polymerization of a covalent organic framework layer on titanium dioxide@ graphene oxide/polyacrylonitrile mixed-matrix membranes for high-performance dye separation. J. Membr. Sci..

[12] Tong H., Liu Q., Xu N., Wang Q., Fan L., Dong Q., Ding A. (2023). Efficient Pervaporation for Ethanol Dehydration: Ultrasonic Spraying Preparation of Polyvinyl Alcohol (PVA)/Ti_3_C_2_Tx Nanosheet Mixed Matrix Membranes. Membranes.

[13] Wang Q., Zhou R., Tsuru T. (2022). Recent Progress in Silicon Carbide-Based Membranes for Gas Separation. Membranes.

[14] Wang Q., Xu N., Liu Q., Dong Q., Nagasawa H., Kanezashi M., Zhou R., Tsuru T. (2022). Low-temperature cross-linking fabrication of sub-nanoporous SiC-based membranes for application to the pervaporation removal of methanol. J. Membr. Sci..

[15] Yu X., Wang Q., Nagasawa H., Kanezashi M., Tsuru T. (2020). SiC mesoporous membranes for sulfuric acid decomposition at high temperatures in the iodine–sulfur process. RSC Adv..

[16] Shao J., Ge Q., Shan L., Wang Z., Yan Y. (2011). Influences of seeds on the properties of zeolite NaA membranes on alumina hollow fibers. Ind. Eng. Chem. Res..

[17] Zhou C., Zhou J., Huang A. (2016). Seeding-free synthesis of zeolite FAU membrane for seawater desalination by pervaporation. Microporous Mesoporous Mater..

[18] Zhou J., Zhou C., Xu K., Caro J., Huang A. (2020). Seeding-free synthesis of large tubular zeolite FAU membranes for dewatering of dimethyl carbonate by pervaporation. Microporous Mesoporous Mater..

[19] Zhou C., Yuan C., Zhu Y., Caro J., Huang A. (2015). Facile synthesis of zeolite FAU molecular sieve membranes on bio-adhesive polydopamine modified Al_2_O_3_ tubes. J. Membr. Sci..

[20] Xia B., Wang S., Li B., Cao Y., Liu T., Gao P., Chen C., Li Y. (2021). Seeding-free synthesis of FAU-type membrane with dry gel modified α-alumina support. Microporous Mesoporous Mater..

[21] Nazir L.S.M., Yeong Y.F., Chew T.L. (2020). Methods and synthesis parameters affecting the formation of FAU type zeolite membrane and its separation performance: A review. J. Asian Ceram. Soc..

[22] Nazir L.S.M., Yeong Y.F., Chew T.L. (2021). Study on the effect of seed particle size toward the formation of NaX zeolite membranes via vacuum-assisted seeding technique. J. Asian Ceram. Soc..

[23] Gu X., Dong J., Nenoff T.M. (2005). Synthesis of defect-free FAU-type zeolite membranes and separation for dry and moist CO_2_/N_2_ mixtures. Ind. Eng. Chem. Res..

[24] Holmberg B.A., Wang H., Norbeck J.M., Yan Y. (2003). Controlling size and yield of zeolite Y nanocrystals using tetramethylammonium bromide. Microporous Mesoporous Mater..

[25] Wang Q., Yu L., Nagasawa H., Kanezashi M., Tsuru T. (2020). High-performance molecular-separation ceramic membranes derived from oxidative cross-linked polytitanocarbosilane. J. Am. Ceram. Soc..

[26] Wang Q., Kawano Y., Yu L., Nagasawa H., Kanezashi M., Tsuru T. (2020). Development of high-performance sub-nanoporous SiC-based membranes derived from polytitanocarbosilane. J. Membr. Sci..

[27] Wang Q., Yu L., Nagasawa H., Kanezashi M., Tsuru T. (2020). Tuning the microstructure of polycarbosilane-derived SiC(O) separation membranes via thermal-oxidative cross-linking. Sep. Purif. Technol..

[28] Wang Q., Yokoji M., Nagasawa H., Yu L., Kanezashi M., Tsuru T. (2020). Microstructure evolution and enhanced permeation of SiC membranes derived from allylhydridopolycarbosilane. J. Membr. Sci..

[29] Zhou R., Zhang Q., Shao J., Wang Z., Chen X., Kita H. (2012). Optimization of NaY zeolite membrane preparation for the separation of methanol/methyl methacrylate mixtures. Desalination.

[30] Kumakiri I., Yamaguchi T., Nakao S.-I. (1999). Preparation of zeolite A and faujasite membranes from a clear solution. Ind. Eng. Chem. Res..

[31] Zhu F., Landon J., Liu K. (2020). FAU zeolite membranes for dewatering of amine-based post-combustion CO_2_ capture solutions. AIChE J..

[32] Okamoto K.I., Kita H., Horii K., Kondo K.T. (2001). Zeolite NaA membrane: Preparation, single-gas permeation, and pervaporation and vapor permeation of water/organic liquid mixtures. Ind. Eng. Chem. Res..

[33] Lucero J.M., Crawford J.M., Wolden C.A., Carreon M.A. (2021). Tunability of ammonia adsorption over NaP zeolite. Microporous Mesoporous Mater..

[34] Lang W.-Z., Ouyang J.-X., Guo Y.-J., Chu L.-F. (2011). Synthesis of tubular faujasite X-type membranes with mullite supports and their gas permeances for N_2_/CO_2_ mixtures. Sep. Sci. Technol..

[35] Wang B., Sun C., Zhou R., Xing W. (2022). A super-permeable and highly-oriented SAPO-34 thin membrane prepared by a green gel-less method using high-aspect-ratio nanosheets for efficient CO_2_ capture. Chem. Eng. J..

[36] Zhang H., Yang Y., Wang Z. (2023). Synthesis of hierarchical LTA zeolite membranes by vapor phase transformation. J. Membr. Sci..

[37] Mundstock A., Wang N., Friebe S., Caro J. (2015). Propane/propene permeation through Na-X membranes: The interplay of separation performance and pre-synthetic support functionalization. Microporous Mesoporous Mater..

[38] Van Miltenburg A., Gascon J., Zhu W., Kapteijn F., Moulijn J.A. (2008). Propylene/propane mixture adsorption on faujasite sorbents. Adsorption.

[39] Carter J.H., Bere T., Pitchers J.R., Hewes D.G., Vandegehuchte B.D., Kiely C.J., Taylor S.H., Hutchings G.J. (2021). Direct and oxidative dehydrogenation of propane: From catalyst design to industrial application. Green Chem..

[40] Ishii K., Shibata A., Takeuchi T., Yoshiura J., Urabe T., Kameda Y., Nomura M. (2019). Development of Silica Membranes to Improve Dehydration Reactions. J. Jpn. Pet. Inst..

[41] Kim S.-J., Tan S., Claure M.T., Gil L.B., More K.L., Liu Y., Moore J.S., Dixit R.S., Pendergast J.G., Sholl D.S. (2016). One-Step Synthesis of Zeolite Membranes Containing Catalytic Metal Nanoclusters. ACS Appl. Mater. Interfaces.

[42] Yang S., Kwon Y.H., Koh D., Min B., Liu Y., Nair S. (2018). Highly Selective SSZ-13 Zeolite Hollow Fiber Membranes by Ultraviolet Activation at Near-Ambient Temperature. Chemnanomat.

[43] Weyten H., Keizer K., Kinoo A., Luyten J., Leysen R. (1997). Dehydrogenation of propane using a packed-bed catalytic membrane reactor. AIChE J..

[44] Wei X.-L., Liu H., Xu Y.-Y., Sun Y.-L., Chao Z.-S. (2018). Synthesis of NaA zeolite membrane by maintaining pressure difference between the two sides of the support. CrystEngComm.

[45] Liu B.S., Au C.T. (2002). Preparation and Separation Performance of a TPAOH-Induced ANA Zeolite Membrane. Chem. Lett..

[46] Morón F., Pina M., Urriolabeitia E., Menéndez M., Santamaría J. (2002). Preparation and characterization of Pd-zeolite composite membranes for hydrogen separation. Desalination.

[47] Kim S.-J., Liu Y., Moore J.S., Dixit R.S., Pendergast J.G., Sholl D., Jones C.W., Nair S. (2016). Thin Hydrogen-Selective SAPO-34 Zeolite Membranes for Enhanced Conversion and Selectivity in Propane Dehydrogenation Membrane Reactors. Chem. Mater..

[48] Tanaka K., Taguchi A., Hao J., Kita H., Okamoto K. (1996). Permeation and separation properties of polyimide membranes to olefins and paraffins. J. Membr. Sci..

[49] Krol J., Boerrigter M., Koops G. (2001). Polyimide hollow fiber gas separation membranes: Preparation and the suppression of plasticization in propane/propylene environments. J. Membr. Sci..

[50] Chan S.S., Wang R., Chung T.S., Liu Y. (2002). C2 and C3 hydrocarbon separations in poly (1, 5-naphthalene-2, 2′-bis (3, 4-phthalic) hexafluoropropane) diimide (6FDA-1, 5-NDA) dense membranes. J. Membr. Sci..

[51] Yoshino M., Nakamura S., Kita H., Okamoto K.-I., Tanihara N., Kusuki Y. (2003). Olefin/paraffin separation performance of asymmetric hollow fiber membrane of 6FDA/BPDA–DDBT copolyimide. J. Membr. Sci..

[52] Swaidan R.J., Ghanem B., Swaidan R., Litwiller E., Pinnau I. (2015). Pure- and mixed-gas propylene/propane permeation properties of spiro- and triptycene-based microporous polyimides. J. Membr. Sci..

[53] Zhang C., Dai Y., Johnson J.R., Karvan O., Koros W.J. (2012). High performance ZIF-8/6FDA-DAM mixed matrix membrane for propylene/propane separations. J. Membr. Sci..

[54] Gajbhiye S.B. (2015). Membranes of benzene sulfonylated-polyphenylene oxide as affinity membranes for propylene and propane gases. Indian J. Chem. Technol..

[55] Staudt-Bickel C., Koros W.J. (2000). Olefin/paraffin gas separations with 6FDA-based polyimide membranes. J. Membr. Sci..

[56] Lin H., Freeman B.D. (2004). Gas solubility, diffusivity and permeability in poly (ethylene oxide). J. Membr. Sci..

[57] Du N., Cin M.M.D., Pinnau I., Nicalek A., Robertson G.P., Guiver M.D. (2011). Azide-based Cross-Linking of Polymers of Intrinsic Microporosity (PIMs) for Condensable Gas Separation. Macromol. Rapid Commun..

[58] Staudt-Bickel C. (2003). Cross-linked Copolyimide Membranes for the Separation of Gaseous and Liquid Mixtures. Soft Mater..

[59] Chen J.C., Feng X., Penlidis A. (2005). Gas permeation through poly (Ether-b-amide)(PEBAX 2533) block copolymer membranes. Sep. Sci. Technol..

[60] Sakai M., Sasaki Y., Tomono T., Seshimo M., Matsukata M. (2019). Olefin Selective Ag-Exchanged X-Type Zeolite Membrane for Propylene/Propane and Ethylene/Ethane Separation. ACS Appl. Mater. Interfaces.

[61] Tiscornia I., Irusta S., Téllez C., Coronas J., Santamaría J. (2007). Separation of propylene/propane mixtures by titanosilicate ETS-10 membranes prepared in one-step seeded hydrothermal synthesis. J. Membr. Sci..

[62] Nikolakis V., Xomeritakis G., Abibi A., Dickson M., Tsapatsis M., Vlachos D.G. (2001). Growth of a faujasite-type zeolite membrane and its application in the separation of saturated/unsaturated hydrocarbon mixtures. J. Membr. Sci..

[63] Giannakopoulos I.G., Nikolakis V. (2004). Separation of Propylene/Propane Mixtures Using Faujasite-Type Zeolite Membranes. Ind. Eng. Chem. Res..

[64] Menendez I., Fuertes A.B. (2001). Aging of carbon membranes under different environments. Carbon.

[65] Fuertes A.B., Menendez I. (2002). Separation of hydrocarbon gas mixtures using phenolic resin-based carbon membranes. Sep. Purif. Technol..

[66] Yoshino M., Nakamura S., Kita H., Okamoto K.-I., Tanihara N., Kusuki Y. (2003). Olefin/paraffin separation performance of carbonized membranes derived from an asymmetric hollow fiber membrane of 6FDA/BPDA–DDBT copolyimide. J. Membr. Sci..

[67] Centeno T., Vilas J., Fuertes A. (2004). Effects of phenolic resin pyrolysis conditions on carbon membrane performance for gas separation. J. Membr. Sci..

[68] Islam N., Zhou W., Honda T., Tanaka K., Kita H., Okamoto K.-I. (2005). Preparation and gas separation performance of flexible pyrolytic membranes by low-temperature pyrolysis of sulfonated polyimides. J. Membr. Sci..

[69] Chng M.L., Xiao Y., Chung T.S., Toriida M., Tamai S. (2009). Enhanced propylene/propane separation by carbonaceous membrane derived from poly (aryl ether ketone)/2, 6-bis (4-azidobenzylidene)-4-methyl-cyclohexanone interpenetrating network. Carbon.

[70] Ma X., Lin B.K., Wei X., Kniep J., Lin Y.S. (2013). Gamma-Alumina Supported Carbon Molecular Sieve Membrane for Propylene/Propane Separation. Ind. Eng. Chem. Res..

[71] Ibrahim S.M., Xu R., Nagasawa H., Naka A., Ohshita J., Yoshioka T., Kanezashi M., Tsuru T. (2014). A closer look at the development and performance of organic–inorganic membranes using 2, 4, 6-tris [3 (triethoxysilyl)-1-propoxyl]-1, 3, 5-triazine (TTESPT). RSC Adv..

[72] Ma X., Williams S., Wei X., Kniep J., Lin Y. (2015). Propylene/Propane Mixture Separation Characteristics and Stability of Carbon Molecular Sieve Membranes. Ind. Eng. Chem. Res..

[73] Swaidan R.J., Ma X., Pinnau I. (2016). Spirobisindane-based polyimide as efficient precursor of thermally-rearranged and carbon molecular sieve membranes for enhanced propylene/propane separation. J. Membr. Sci..

[74] Ma X., Lin Y.S., Wei X., Kniep J. (2015). Ultrathin carbon molecular sieve membrane for propylene/propane separation. AIChE J..

[75] Liu J., Xiao Y., Chung T.-S. (2017). Flexible thermally treated 3D PIM-CD molecular sieve membranes exceeding the upper bound line for propylene/propane separation. J. Mater. Chem. A.

[76] Kim S.J., Lee P.S., Chang J.S., Nam S.E., Park Y.I. (2018). Preparation of carbon molecular sieve membranes on low-cost alumina hollow fibers for use in C3H6/C3H8 separation. Sep. Purif. Technol..

[77] Karunaweera C., Musselman I.H., Balkus K.J., Ferraris J.P. (2019). Fabrication and characterization of aging resistant carbon molecular sieve membranes for C3 separation using high molecular weight crosslinkable polyimide, 6FDA-DABA. J. Membr. Sci..

[78] Zhang C., Zhang K., Xu L., Labreche Y., Kraftschik B., Koros W.J. (2014). Highly scalable ZIF-based mixed-matrix hollow fiber membranes for advanced hydrocarbon separations. AIChE J..

[79] Sun H., Ma C., Wang T., Xu Y., Yuan B., Li P., Kong Y. (2014). Preparation and Characterization of C_60_-Filled Ethyl Cellulose Mixed-Matrix Membranes for Gas Separation of Propylene/Propane. Chem. Eng. Technol..

[80] Naghsh M., Sadeghi M., Moheb A., Chenar M.P., Mohagheghian M. (2012). Separation of ethylene/ethane and propylene/propane by cellulose acetate–silica nanocomposite membranes. J. Membr. Sci..

[81] Ahmadizadegan H., Ghavvas F., Ranjbar M., Esmaielzadeh S. (2017). Synthesis and characterization of fluorinated polyimide/TiO2 nanocomposites: Enhancement of separation of four gases, thermal, optical and mechanical properties. Polym. Bull..

[82] Davoodi S.M., Sadeghi M., Naghsh M., Moheb A. (2016). Olefin–paraffin separation performance of polyimide Matrimid^®^/silica nanocomposite membranes. RSC Adv..

[83] Askari M., Chung T.-S. (2013). Natural gas purification and olefin/paraffin separation using thermal cross-linkable co-polyimide/ZIF-8 mixed matrix membranes. J. Membr. Sci..

[84] Liu Y., Chen Z., Liu G., Belmabkhout Y., Adil K., Eddaoudi M., Koros W. (2019). Conformation-Controlled Molecular Sieving Effects for Membrane-Based Propylene/Propane Separation. Adv. Mater..

[85] Ma X., Swaidan R.J., Wang Y., Hsiung C.-E., Han Y., Pinnau I. (2018). Highly Compatible Hydroxyl-Functionalized Microporous Polyimide-ZIF-8 Mixed Matrix Membranes for Energy Efficient Propylene/Propane Separation. ACS Appl. Nano Mater..

[86] Liu D., Xiang L., Chang H., Chen K., Wang C., Pan Y., Li Y., Jiang Z. (2019). Rational matching between MOFs and polymers in mixed matrix membranes for propylene/propane separation. Chem. Eng. Sci..

[87] Amedi H.R., Aghajani M. (2018). Poly urethane mixed matrix membranes for propylene and propane separation. Chem. Pap..

[88] Jung J.P., Kim M.J., Bae Y.S., Kim J.H. (2018). Facile preparation of Cu (I) impregnated MIL-101 (Cr) and its use in a mixed matrix membrane for olefin/paraffin separation. J. Appl. Polym. Sci..

[89] Amedi H.R., Aghajani M. (2018). Economic Estimation of Various Membranes and Distillation for Propylene and Propane Separation. Ind. Eng. Chem. Res..

[90] An H., Park S., Kwon H.T., Jeong H.-K., Lee J.S. (2017). A new superior competitor for exceptional propylene/propane separations: ZIF-67 containing mixed matrix membranes. J. Membr. Sci..

[91] Shen Q., Cong S., He R., Wang Z., Jin Y., Li H., Cao X., Wang J., Van der Bruggen B., Zhang Y. (2019). SIFSIX-3-Zn/PIM-1 mixed matrix membranes with enhanced permeability for propylene/propane separation. J. Membr. Sci..

