# Supplementary material for: High-Performance FAU Zeolite Membranes Derived from Nano-Seeds for Gas Separation
Source: Membranes (Basel). 2023 Oct 26;13(11):858. doi: 10.3390/membranes13110858 (PMC10672818; doi:10.3390/membranes13110858)
Supplement: Supplementary file 1 [file membranes-13-00858-s001.zip › membranes-2670916-supplementary.pdf]

membranes

MDPI

Supporting information

High-Performance FAU Zeolite Membranes Derived from Nano-Seeds for Gas Separation

Qing Wang[1,2,*], Huiyuan Chen[1], Feiyang He[1], Qiao Liu[1], Nong Xu[1], Long Fan[1], Chuyan Wang[3], Lingyun Zhang[1] and Rongfei Zhou[2,*]

1. School of Energy, Materials and Chemical Engineering, Hefei University, Hefei 230601, China
2. State Key Laboratory of Materials-Oriented Chemical Engineering, College of Chemical Engineering, Nanjing Tech University, Nanjing 210009, China
3. School of Biological Food and Environment, Hefei University, Hefei 230601, China

* Correspondence: qingwang@hfuu.edu.cn (Q.W.); rf-zhou@njtech.edu.cn (R.F.Z.)

Table S1 Comparison of the H_2/C_3H_8 separation properties of FAU membranes (MA and MM) with other membranes from recent literature.

Membrane type	Temperature [°C]	H_2 permeance [mol/(m^2 s Pa)]	H_2/C_3H_8 selectivity [-]	Ref.
Silica	500	1.00E-07	98	[1]
	650	1.60E-07	111	
Silica	500	1.57E-07	139	
	650	2.78E-07	201	
MFI	25	7.26E-08	0.86	[2]
	100	9.20E-08	0.86	
	200	1.82E-07	1.21	
	300	2.06E-07	1.54	
	400	2.28E-07	2.48	
	450	2.28E-07	3.33	
	500	2.29E-07	3.99	
	550	2.36E-07	5.17	
	600	2.56E-07	6.34	
	650	2.56E-07	6.88	
SSZ-13	25	7.10E-06	4	[3]
	25	3.20E-06	4	
	25	6.60E-08	520	
	25	6.00E-08	590	
	25	8.40E-08	810	
	25	3.00E-08	220	
Silica	200	1.53E-08	4.6	[4]
	200	1.66E-08	3.7	
	400	3.13E-08	14	
	400	3.31E-08	11	
	500	1.39E-07	92	
	500	1.43E-07	67	
NaA	25	1.70E-05	3.95	[5]
	25	3.61E-06	11.34	
ANA Zeolite	25	1.07E-06	138	[6]
NaA	50	4.81E-07	7.28	[7]
	100	3.44E-07	5.72	
	250	1.62E-07	4.82	
	100	2.06E-07	4.62	
	200	2.46E-07	4.02	
	300	4.45E-07	4.38	

SAPO-34	25	4.20E-08	18	[8]
	25	4.80E-08	21	
	25	8.60E-08	18	
	25	1.12E-07	16	
	25	1.23E-07	22	
	25	4.77E-08	5	
	100	5.73E-08	7	
	200	7.16E-08	10	
	300	9.36E-08	14	
	400	1.16E-07	14	
	450	1.37E-07	16	
	500	1.52E-07	19	
	550	1.87E-07	22	
	600	2.01E-07	23	
	650	2.30E-07	27	
NaY	Room temperature	5.34E-07	183	This work

Table S2. Membrane separation performance data of C_3H_6/C_3H_8

Material	Permeability/ Barrer	Selectivity	Membrane type	Reference
6FDA-TeMPD	37	8.6	Polymers	[9]
6FDA-TrMPD	30	11	Polymers	[9]
6FDA-DDBT	0.76	27	Polymers	[9]
6FDA-ODA	0.48	11	Polymers	[9]
BPDA-TeMPD	3.2	13	Polymers	[9]
PPO	2.9	9.1	Polymers	[9]
P4MP	54	2	Polymers	[9]
1.2PB	260	1.7	Polymers	[9]
PDMS	6600	1.1	Polymers	[9]
Matrimid® 5218	0.1	16	Polymers	[10]
6FDA-1,5-NDA	0.24	5.8	Polymers	[11]
6FDA-BPDA/DDBT(1/1)	1	31	Polymers	[12]
PIM-PIs	393	6	Polymers	[13]
6FDA-DAM	15.7	12.4	Polymers	[14]
sulfonylated PPO	18.1	3.05	Polymers	[15]
6FDA-based PI	0.89	16	Polymers	[16]
PEO	6.6	2.75	Polymers	[17]
P84/Matrimid	7	18.2	Polymers	[17]
azide-PIM	40	10	Polymers	[18]
crosslinked PI	4.5	11	Polymers	[19]
Pebax 2533	580	1.6	Polymers	[20]
Na-X-Ag	420	50	Zeolites	[21]
ETS-10	944	5.5	Zeolites	[22]
Zeolite Y-Ag	1016	4.8	Zeolites	[23]
Na-X	520	6.2	Zeolites	[24]
Na-X	428	13.7	Zeolites	[25]
Na-X	420	3.3	Zeolites	[26]
NaY	1090	46	Zeolites	This Work
Phenolic resin carbon membrane	90	17	Carbon Membranes	[27]
Phenolic resin carbon membrane	200	3.1	Carbon Membranes	[28]

PI-based carbon membrane	5.2	22	Carbon Membranes	[29]
Phenolic resin carbon membrane	19	5.4	Carbon Membranes	[30]
Sulfonated PI-based carbon membranes	15	4.2	Carbon Membranes	[31]
Interpenetrating network-based carbon membrane	48	44	Carbon Membranes	[32]
PI-based carbon membrane	14.4	36	Carbon Membranes	[33]
Silicon membrane	13.3	37	Carbon Membranes	[34]
PI-based carbon membrane	0.528	35	Carbon Membranes	[35]
PIM-PI-based carbon membrane	45	33	Carbon Membranes	[36]
PI-based carbon membrane	15.6	31	Carbon Membranes	[37]
PIM-β-CD	1500	5.8	Carbon Membranes	[38]
PI-based carbon membrane	240	30	Carbon Membranes	[39]
PI-based carbon membrane	403	25	Carbon Membranes	[40]
6FDA-DAM/ZIF-8	100	6.6	Mixed Matrix Membranes	[41]
Ethy CA/C_{60}	61.3	4.9	Mixed Matrix Membranes	[42]
CA/SiO_2	0.098	6.12	Mixed Matrix Membranes	[43]
Fluorinated PI/TiO_2	0.08	5.11	Mixed Matrix Membranes	[44]

Matrimid/SiO_2	0.16	18.03	Mixed Matrix Membranes	[45]
Thermal crosslinked PI/ZIF-8	42.7	27.47	Mixed Matrix Membranes	[46]
6FDA-DAM+Y-fum- fcu-MOF	33.4	18	Mixed Matrix Membranes	[47]
PIM-6FDA-OH/ZIF-8	38	43	Mixed Matrix Membranes	[48]
XLPEO/ZIF-8	28	15	Mixed Matrix Membranes	[49]
PU/ZIF-8	140	3.5	Mixed Matrix Membranes	[50]
SBS/Cu@MIL-101(Cr)	353	1.9	Mixed Matrix Membranes	[51]
Pebax 1657/ZIF-8	84	5.57	Mixed Matrix Membranes	[52]
6FDA-DAM/ZIF-8	56.2	31	Mixed Matrix Membranes	[14]
6FDA-DAM/ZIF-67	34.1	29.9	Mixed Matrix Membranes	[53]
PIM-1/SIFSIX-3-Zn	4012	7.9	Mixed Matrix Membranes	[54]

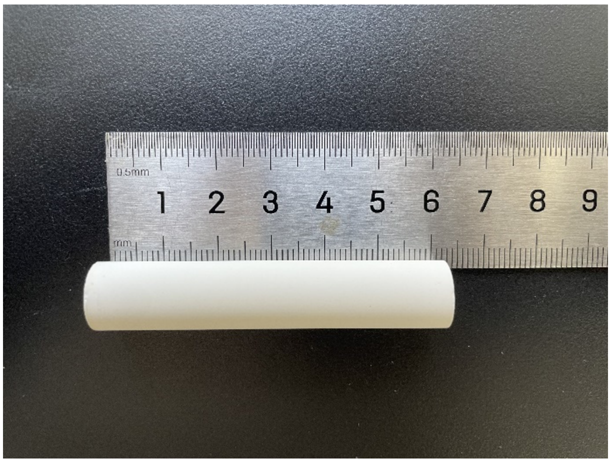

Figure S1. The optical photograph of an FAU (MA) membrane.

References:

1. Ishii, K.; Shibata, A.; Takeuchi, T.; Yoshiura, J.; Urabe, T.; Kameda, Y.; Nomura, M. Development of Silica Membranes to Improve Dehydration Reactions. *J. Jpn. Pet. Inst.* **2019**, *62,* 211–219.
2. Kim, S.-J.; Tan, S.; Claure, M.T.; Gil, L.B.; More, K.L.; Liu, Y.; Moore, J.S.; Dixit, R.S.; Pendergast, J.G.; Sholl, D.S.; et al. One-Step Synthesis of Zeolite Membranes Containing Catalytic Metal Nanoclusters. *ACS Appl. Mater. Interfaces* **2016**, *8*, 24671–24681, https://doi.org/10.1021/acsami.6b06576.
3. Yang, S.; Kwon, Y.H.; Koh, D.; Min, B.; Liu, Y.; Nair, S. Highly Selective SSZ-13 Zeolite Hollow Fiber Membranes by Ultraviolet Activation at Near-Ambient Temperature. *Chemnanomat* **2018**, *5*, 61–67, https://doi.org/10.1002/cnma.201800272.
4. Weyten, H.; Keizer, K.; Kinoo, A.; Luyten, J.; Leysen, R. Dehydrogenation of propane using a packed-bed catalytic membrane reactor. *AIChE J.* **1997**, *43*, 1819–1827, https://doi.org/10.1002/aic.690430717.
5. Wei, X.-L.; Liu, H.; Xu, Y.-Y.; Sun, Y.-L.; Chao, Z.-S. Synthesis of NaA zeolite membrane by maintaining pressure difference between the two sides of the support. *CrystEngComm* **2018**, *20*, 7195–7205, https://doi.org/10.1039/c8ce01175c.
6. Liu, B.S.; Au, C.T. Preparation and Separation Performance of a TPAOH-Induced ANA Zeolite Membrane. *Chem. Lett.* **2002**, *31*, 806–807, https://doi.org/10.1246/cl.2002.806.
7. Morón, F.; Pina, M.; Urriolabeitia, E.; Menéndez, M.; Santamaría, J. Preparation and characterization of Pd-zeolite composite membranes for hydrogen separation. *Desalination* **2002**, *147*, 425–431, https://doi.org/10.1016/s0011-9164(02)00638-0.
8. Kim, S.-J.; Liu, Y.; Moore, J.S.; Dixit, R.S.; Pendergast, J.G.; Sholl, D.; Jones, C.W.; Nair, S. Thin Hydrogen-Selective SAPO-34 Zeolite Membranes for Enhanced Conversion and Selectivity in Propane Dehydrogenation Membrane Reactors. *Chem. Mater.* **2016**, *28*, 4397–4402, https://doi.org/10.1021/acs.chemmater.6b01458.
9. Tanaka, K.; Taguchi, A.; Hao, J.; Kita, H.; Okamoto, K. Permeation and separation properties of polyimide membranes to olefins and paraffins. *J. Membr. Sci.* **1996**, *121*, 197–207, https://doi.org/10.1016/s0376-7388(96)00182-2.
10. Krol, J.; Boerrigter, M.; Koops, G. Polyimide hollow fiber gas separation membranes: preparation and the suppression of plasticization in propane/propylene environments. *J. Membr. Sci.* **2001**, *184*, 275–286, https://doi.org/10.1016/s0376-7388(00)00640-2.
11. Chan, S.S., Wang, R., Chung, T.S. and Liu, Y. C2 and C3 hydrocarbon separations in poly (1, 5-naphthalene-2, 2′-bis (3, 4-phthalic) hexafluoropropane) diimide (6FDA-1, 5-NDA) dense membranes. *J. Membr. Sci.* **2002**, *210*, 55–64.
12. Yoshino, M.; Nakamura, S.; Kita, H.; Okamoto, K.-I.; Tanihara, N.; Kusuki, Y. Olefin/paraffin separation performance of asymmetric hollow fiber membrane of 6FDA/BPDA–DDBT copolyimide. *J. Membr. Sci.* **2003**, *212*, 13–27, https://doi.org/10.1016/s0376-7388(02)00434-9.
13. Swaidan, R.J.; Ghanem, B.; Swaidan, R.; Litwiller, E.; Pinnau, I. Pure- and mixed-gas propylene/propane permeation properties of spiro- and triptycene-based microporous polyimides. *J. Membr. Sci.* **2015**, *492*, 116–122, https://doi.org/10.1016/j.memsci.2015.05.044.
14. Zhang, C., Dai, Y., Johnson, J.R., Karvan, O. and Koros, W.J. High performance ZIF-8/6FDA-DAM mixed matrix membrane for propylene/propane separations. *J. Membr. Sci.* **2012**, *389*, 34–42.
15. Gajbhiye, S.B. Membranes of benzene sulfonylated-polyphenylene oxide as affinity membranes for propylene and propane gases. *Indian J. Chem. Technol.* **2015**, *22*, 105–112.
16. Staudt-Bickel, C.; Koros, W.J. Olefin/paraffin gas separations with 6FDA-based polyimide membranes. *J. Membr. Sci.* **2000**, *170*, 205–214, https://doi.org/10.1016/s0376-7388(99)00351-8.
17. Lin, H. and Freeman, B.D. Gas solubility, diffusivity and permeability in poly (ethylene oxide). *J. Membr. Sci.* **2004**, *239*, 105–117.

18. Du, N.; Cin, M.M.D.; Pinnau, I.; Nicalek, A.; Robertson, G.P.; Guiver, M.D. Azide-based Cross-Linking of Polymers of Intrinsic Microporosity (PIMs) for Condensable Gas Separation. *Macromol. Rapid Commun.* **2011**, *32*, 631–636, https://doi.org/10.1002/marc.201000775.
19. Staudt-Bickel, C. Cross-linked Copolyimide Membranes for the Separation of Gaseous and Liquid Mixtures. *Soft Mater.* **2003**, *1*, 277–293, https://doi.org/10.1081/smts-120026594.
20. Chen, J.C., Feng, X. and Penlidis, A. Gas permeation through poly (Ether-b-amide)(PEBAX 2533) block copolymer membranes, *Sep. Sci. Technol.* **2005**, *39*, 149–164.
21. Sakai, M.; Sasaki, Y.; Tomono, T.; Seshimo, M.; Matsukata, M. Olefin Selective Ag-Exchanged X-Type Zeolite Membrane for Propylene/Propane and Ethylene/Ethane Separation. *ACS Appl. Mater. Interfaces* **2019**, *11*, 4145–4151, https://doi.org/10.1021/acsami.8b20151.
22. Tiscornia, I.; Irusta, S.; Téllez, C.; Coronas, J.; Santamaría, J. Separation of propylene/propane mixtures by titanosilicate ETS-10 membranes prepared in one-step seeded hydrothermal synthesis. *J. Membr. Sci.* **2007**, *311*, 326–335, https://doi.org/10.1016/j.memsci.2007.12.028.
23. Shrestha, S.; Dutta, P.K. Modification of a continuous zeolite membrane grown within porous polyethersulfone with Ag(I) cations for enhanced propylene/propane gas separation. *Microporous Mesoporous Mater.* **2018**, *279*, 178–185, https://doi.org/10.1016/j.micromeso.2018.12.032.
24. Nikolakis, V.; Xomeritakis, G.; Abibi, A.; Dickson, M.; Tsapatsis, M.; Vlachos, D.G. Growth of a faujasite-type zeolite membrane and its application in the separation of saturated/unsaturated hydrocarbon mixtures. *J. Membr. Sci.* **2001**, *184*, 209–219, https://doi.org/10.1016/s0376-7388(00)00623-2.
25. Giannakopoulos, I.G.; Nikolakis, V. Separation of Propylene/Propane Mixtures Using Faujasite-Type Zeolite Membranes. *Ind. Eng. Chem. Res.* **2004**, *44*, 226–230, https://doi.org/10.1021/ie049508r.
26. Mundstock, A.; Wang, N.; Friebe, S.; Caro, J. Propane/propene permeation through Na-X membranes: The interplay of separation performance and pre-synthetic support functionalization. *Microporous Mesoporous Mater.* **2015**, *215*, 20–28, https://doi.org/10.1016/j.micromeso.2015.05.019.
27. Menendez, I.; Fuertes, A.B. Aging of carbon membranes under different environments. *Carbon* **2001**, *39*, 733–740, https://doi.org/10.1016/s0008-6223(00)00188-3.
28. Fuertes, A.B.; Menendez, I. Separation of hydrocarbon gas mixtures using phenolic resin-based carbon membranes. *Sep. Purif. Technol.* **2002**, *28*, 29–41, https://doi.org/10.1016/s1383-5866(02)00006-0.
29. Yoshino, M.; Nakamura, S.; Kita, H.; Okamoto, K.-I.; Tanihara, N.; Kusuki, Y. Olefin/paraffin separation performance of carbonized membranes derived from an asymmetric hollow fiber membrane of 6FDA/BPDA–DDBT copolyimide. *J. Membr. Sci.* **2003**, *215*, 169–183, https://doi.org/10.1016/s0376-7388(02)00611-7.
30. Centeno, T.; Vilas, J.; Fuertes, A. Effects of phenolic resin pyrolysis conditions on carbon membrane performance for gas separation. *J. Membr. Sci.* **2004**, *228*, 45–54, https://doi.org/10.1016/j.memsci.2003.09.010.
31. Islam, N.; Zhou, W.; Honda, T.; Tanaka, K.; Kita, H.; Okamoto, K.-I. Preparation and gas separation performance of flexible pyrolytic membranes by low-temperature pyrolysis of sulfonated polyimides. *J. Membr. Sci.* **2005**, *261*, 17–26, https://doi.org/10.1016/j.memsci.2005.02.019.
32. Chng, M.L., Xiao, Y., Chung, T.S., Toriida, M. and Tamai, S. Enhanced propylene/propane separation by carbonaceous membrane derived from poly (aryl ether ketone)/2, 6-bis (4-azidobenzylidene)-4-methyl-cyclohexanone interpenetrating network. *Carbon* **2009**, *47*, 1857–1866.
33. Ma, X.; Lin, B.K.; Wei, X.; Kniep, J.; Lin, Y.S. Gamma-Alumina Supported Carbon Molecular Sieve Membrane for Propylene/Propane Separation. *Ind. Eng. Chem. Res.* **2013**, *52*, 4297–4305, https://doi.org/10.1021/ie303188c.
34. Ibrahim, S.M., Xu, R., Nagasawa, H., Naka, A., Ohshita, J., Yoshioka, T., Kanezashi, M. and Tsuru, T. A closer look at the development and performance of organic–inorganic membranes using 2, 4, 6-tris [3 (triethoxysilyl)-1-propoxyl]-1, 3, 5-triazine (TTESPT), *RSC Adv.* **2014**, *4*, 12404–12407.
35. Ma, X.; Williams, S.; Wei, X.; Kniep, J.; Lin, Y. Propylene/Propane Mixture Separation Characteristics and Stability of Carbon Molecular Sieve Membranes. *Ind. Eng. Chem. Res.* **2015**, *54*, 9824–9831, https://doi.org/10.1021/acs.iecr.5b02721.
36. Swaidan, R.J.; Ma, X.; Pinnau, I. Spirobisindane-based polyimide as efficient precursor of thermally-rearranged and carbon molecular sieve membranes for enhanced propylene/propane separation. *J. Membr. Sci.* **2016**, *520*, 983–989, https://doi.org/10.1016/j.memsci.2016.08.057.
37. Ma, X.; Lin, Y.S.; Wei, X.; Kniep, J. Ultrathin carbon molecular sieve membrane for propylene/propane separation. *AIChE J.* **2015**, *62*, 491–499, https://doi.org/10.1002/aic.15005.
38. Liu, J.; Xiao, Y.; Chung, T.-S. Flexible thermally treated 3D PIM-CD molecular sieve membranes exceeding the upper bound line for propylene/propane separation. *J. Mater. Chem. A* **2017**, *5*, 4583–4595, https://doi.org/10.1039/c6ta09751k.
39. Kim, S.J., Lee, P.S., Chang, J.S., Nam, S.E. and Park, Y.I. Preparation of carbon molecular sieve membranes on low-cost alumina hollow fibers for use in C3H6/C3H8 separation. *Sep. Purif. Technol.* ***2018***, *194*, 443–450.
40. Karunaweera, C.; Musselman, I.H.; Balkus, K.J.; Ferraris, J.P. Fabrication and characterization of aging resistant carbon molecular sieve membranes for C3 separation using high molecular weight crosslinkable polyimide, 6FDA-DABA. *J. Membr. Sci.* **2019**, *581*, 430–438, https://doi.org/10.1016/j.memsci.2019.03.065.

41. Zhang, C.; Zhang, K.; Xu, L.; Labreche, Y.; Kraftschik, B.; Koros, W.J. Highly scalable ZIF-based mixed-matrix hollow fiber membranes for advanced hydrocarbon separations. *AIChE J.* **2014**, *60*, 2625–2635, https://doi.org/10.1002/aic.14496.
42. Sun, H.; Ma, C.; Wang, T.; Xu, Y.; Yuan, B.; Li, P.; Kong, Y. Preparation and Characterization of C_{60}-Filled Ethyl Cellulose Mixed-Matrix Membranes for Gas Separation of Propylene/Propane. *Chem. Eng. Technol.* **2014**, *37*, 611–619, https://doi.org/10.1002/ceat.201300667.
43. Naghsh, M.; Sadeghi, M.; Moheb, A.; Chenar, M.P.; Mohagheghian, M. Separation of ethylene/ethane and propylene/propane by cellulose acetate–silica nanocomposite membranes. *J. Membr. Sci.* **2012**, *423-424*, 97–106, https://doi.org/10.1016/j.memsci.2012.07.032.
44. Ahmadizadegan, H.; Ghavvas, F.; Ranjbar, M.; Esmaielzadeh, S. Synthesis and characterization of fluorinated polyimide/TiO2 nanocomposites: enhancement of separation of four gases, thermal, optical and mechanical properties. *Polym. Bull.* **2017**, *75*, 2729–2750, https://doi.org/10.1007/s00289-017-2179-8.
45. Davoodi, S.M.; Sadeghi, M.; Naghsh, M.; Moheb, A. Olefin–paraffin separation performance of polyimide Matrimid®/silica nanocomposite membranes. *RSC Adv.* **2016**, *6*, 23746–23759, https://doi.org/10.1039/C6RA00553E.
46. Askari, M.; Chung, T.-S. Natural gas purification and olefin/paraffin separation using thermal cross-linkable co-polyimide/ZIF-8 mixed matrix membranes. *J. Membr. Sci.* **2013**, *444*, 173–183, https://doi.org/10.1016/j.memsci.2013.05.016.
47. Liu, Y.; Chen, Z.; Liu, G.; Belmabkhout, Y.; Adil, K.; Eddaoudi, M.; Koros, W. Conformation-Controlled Molecular Sieving Effects for Membrane-Based Propylene/Propane Separation. *Adv. Mater.* **2019**, *31*, e1807513, https://doi.org/10.1002/adma.201807513.
48. Ma, X.; Swaidan, R.J.; Wang, Y.; Hsiung, C.-E.; Han, Y.; Pinnau, I. Highly Compatible Hydroxyl-Functionalized Microporous Polyimide-ZIF-8 Mixed Matrix Membranes for Energy Efficient Propylene/Propane Separation. *ACS Appl. Nano Mater.* **2018**, *1*, 3541–3547, https://doi.org/10.1021/acsanm.8b00682.
49. Liu, D.; Xiang, L.; Chang, H.; Chen, K.; Wang, C.; Pan, Y.; Li, Y.; Jiang, Z. Rational matching between MOFs and polymers in mixed matrix membranes for propylene/propane separation. *Chem. Eng. Sci.* **2019**, *204*, 151–160, https://doi.org/10.1016/j.ces.2019.04.032.
50. Amedi, H.R.; Aghajani, M. Poly urethane mixed matrix membranes for propylene and propane separation. *Chem. Pap.* **2018**, *72*, 1477–1485, https://doi.org/10.1007/s11696-018-0386-x.
51. Jung, J.P., Kim, M.J., Bae, Y.S. and Kim, J.H. Facile preparation of C u (I) impregnated MIL-101 (C r) and its use in a mixed matrix membrane for olefin/paraffin separation. *J. Appl. Polym. Sci.* **2018**, *135*, 46545.
52. Amedi, H.R.; Aghajani, M. Economic Estimation of Various Membranes and Distillation for Propylene and Propane Separation. *Ind. Eng. Chem. Res.* **2018**, *57*, 4366–4376, https://doi.org/10.1021/acs.iecr.7b04169.
53. An, H.; Park, S.; Kwon, H.T.; Jeong, H.-K.; Lee, J.S. A new superior competitor for exceptional propylene/propane separations: ZIF-67 containing mixed matrix membranes. *J. Membr. Sci.* **2017**, *526*, 367–376, https://doi.org/10.1016/j.memsci.2016.12.053.
54. Shen, Q.; Cong, S.; He, R.; Wang, Z.; Jin, Y.; Li, H.; Cao, X.; Wang, J.; Van der Bruggen, B.; Zhang, Y. SIFSIX-3-Zn/PIM-1 mixed matrix membranes with enhanced permeability for propylene/propane separation. *J. Membr. Sci.* **2019**, *588*, 117201, https://doi.org/10.1016/j.memsci.2019.117201.

Disclaimer/Publisher's Note: The statements, opinions and data contained in all publications are solely those of the individual author(s) and contributor(s) and not of MDPI and/or the editor(s). MDPI and/or the editor(s) disclaim responsibility for any injury to people or property resulting from any ideas, methods, instructions or products referred to in the content.
